# On 50th Anniversary of the Global Carbon Dioxide Record

**DOI:** 10.1186/1750-0680-2-11

**Published:** 2007-12-18

**Authors:** Georgii A Alexandrov, Martin Heimann, Chris D Jones, Pieter Tans

**Affiliations:** 1 National Institute for Environmental Studies, Onogawa 16-2, Tsukuba, 305-8506, Japan; 2 Institute of Atmospheric Physics, Russian Academy of Sciences, Pyzhevsky 3, Moscow, 109017, Russia; 3 Max Plank Institute for Biogeochemistry, Jena, Germany; 4 Met Office Hadley Centre, FitzRoy Road, Exeter, EX1 3PB, UK; 5 NOAA Earth System Research Laboratory, 325 Broadway, Boulder, CO 80305, USA

## Abstract

The 50-year global CO_2 _record led the way in establishing a scientific fact: modern civilization is changing important properties of the global atmosphere, oceans and biosphere. The evidence on which this scientific fact is based will be refined further, but the next challenge for scientists is broader. In addition to its traditional role in providing discovery, diagnosis, and prediction of the changes that are taking place on our planet, science has now also a role in helping society mitigate emissions by objectively quantifying them, and in helping adaptation by providing environmental forecasts on regional scales. Science is also expected to provide new options for society to tackle the transition to a new energy system, and to provide thorough environmental evaluation of all such options. This is what the meeting recognized as planetary responsibilities for scientists in the next 50 years.

## Introduction

The ultimate purpose of science is establishing how the Earth system works – concepts that are based on evidence we all can agree on regardless of our religious believes or personal opinions. This typically does not happen in a short time and needs continuous ongoing efforts of dedicated researchers. The 50-year global CO_2 _record [1,2] resulted in established scientific fact: modern civilization is changing the concentration of atmospheric CO_2 _significantly. Other evidence shows the world has not experienced this for a million years, and probably for much longer. It is also well known that the planetary heat budget is significantly changed as a result.

## Discussion

This scientific fact is based on the range of empirical findings. Each of them may need further refinement, and so we listed some of them below to display the current state of knowledge and horizons for further research.

1. The increase of CO_2 _in the atmosphere and in the oceans add up to, within uncertainty, almost the total production of CO_2 _from the burning of fossil fuels [3], showing that enhanced terrestrial biospheric activity has compensated CO_2 _release from anthropogenic land-use changes, such as large scale deforestation. Net terrestrial sources from pre-industrial times showed net emission before 1940, and a small net sink since then [3].

2. The airborne fraction of CO_2 _emission, defined as the observed atmospheric increase divided by emissions of fossil CO_2_, has been remarkably constant over the last 50 years [6]. This is not a fundamental property of the carbon cycle, however [4]. The past record is not sufficient on its own to determine how the airborne fraction will evolve in the future.

3. The inter-annual variations of the growth rate of atmospheric CO_2 _are controlled by the terrestrial biosphere, which exhibits a delayed response to anomalies of both global temperature and precipitation [3]. This provides us with evidence of natural carbon cycle sensitivity to climate, and may provide constraints on future feedbacks in response to long-term climate change.

4. The long-term increase of the amplitude of the seasonal cycle and its interannual variations displays a response of the northern hemisphere terrestrial biosphere to climate variations and trends [5].

5. Based on coupled carbon cycle – climate model simulations, climate feedbacks are expected to amplify global warming over the next hundred years [6,7]. Continued long term monitoring of CO_2 _at Mauna Loa and at other sites is required to uncover more information about the carbon cycle and to reduce the uncertainty in climate feedbacks [4,6,8,9].

6. The observed history of the global carbon cycle together with fundamental process understanding of carbon uptake in the ocean and on land imply that over the next decades emissions have to be reduced to levels less than 50% than today, if atmospheric concentrations are to be stabilized below 550 ppm by the end of this century. Possible positive climate feedbacks imply even stronger emission reductions for CO_2 _concentration stabilization.

## Conclusion

The next generation of scientists will live in a world that is aware of its responsibility for planetary change and will demand globally concerted actions [10,11]. Revealing the effectiveness of these actions in changing the conditions of the disturbed Earth system is one of the things the world community is likely to expect from scientists. Young scientists must be prepared to meet this challenge, which has to take shape in new institutional frameworks [12].
